# Serum uric acid levels and longitudinal change in cognitive function in older adults: a sex-stratified population-based study

**DOI:** 10.1093/gerona/glaf296

**Published:** 2025-12-30

**Authors:** Md Golam Rabbani, Sheikh M Alif, Joanne Ryan, Zhen Zhou, Cammie Tran, Amanda J Rickard, Catherine Robb, Robyn L Woods, Suzanne G Orchard, Raj C Shah, Anne M Murray, John J McNeil, Md Nazmul Karim

**Affiliations:** School of Public Health and Preventive Medicine, Monash University, Melbourne, Victoria, Australia; School of Public Health and Preventive Medicine, Monash University, Melbourne, Victoria, Australia; Institute of Health and Wellbeing, Federation University Australia, Berwick, Victoria, Australia; School of Public Health and Preventive Medicine, Monash University, Melbourne, Victoria, Australia; School of Public Health and Preventive Medicine, Monash University, Melbourne, Victoria, Australia; School of Public Health and Preventive Medicine, Monash University, Melbourne, Victoria, Australia; School of Public Health and Preventive Medicine, Monash University, Melbourne, Victoria, Australia; School of Public Health and Preventive Medicine, Monash University, Melbourne, Victoria, Australia; Industrial Transformation Training Centre for Optimal Ageing, Monash University, Melbourne, Victoria, Australia; Turner Institute for Brain and Mental Health, School of Psychological Sciences, Monash University, Melbourne, Victoria, Australia; School of Public Health and Preventive Medicine, Monash University, Melbourne, Victoria, Australia; School of Public Health and Preventive Medicine, Monash University, Melbourne, Victoria, Australia; Department of Family Medicine and the Rush Alzheimer’s Disease Center, Rush University Medical Center, Chicago, Illinois, United States; Berman Center for Outcomes and Clinical Research, Hennepin Healthcare Research Institute, Minneapolis, Minnesota, United States; Department of Medicine, Geriatrics Division, Hennepin Healthcare, Minneapolis, Minnesota, United States; School of Public Health and Preventive Medicine, Monash University, Melbourne, Victoria, Australia; School of Public Health and Preventive Medicine, Monash University, Melbourne, Victoria, Australia

**Keywords:** Serum uric acid, Cognitive decline, Sex differences, Cognition, Antioxidant

## Abstract

**Background:**

Serum uric acid (SUA) has been linked to cognitive function, but sex-specific associations remain unclear. Biological differences in SUA levels between sexes, driven by hormonal and renal factors, highlight the importance of sex-stratified analysis. This study examined the association between SUA levels and changes in cognitive function in older adults.

**Methods:**

A total of 11 411 community-dwelling ASPirin in Reducing Events in the Elderly participants, free from dementia at baseline and with valid SUA measurements, were included. The Modified Mini-Mental State Examination (3MS), Hopkins Verbal Learning Test–Revised (HVLT-R), Symbol Digit Modalities Test, and Controlled Oral Word Association Test were used to assess cognition at baseline and over a median follow-up of 9 years. Separate linear mixed-effects regression models in males and females were fitted to assess the associations between SUA levels and change in cognitive function over time.

**Results:**

Females in the lowest SUA quintile (Q1) had significant declines in the measure of global cognition (3MS: β ±SE= −0.07 ± 0.03, *p* = .02) and episodic memory (HVLT-R; delayed recall: β±SE= -0.03 ± 0.01, *p* = .02) compared to the middle quintiles (Q2-Q4), but the highest SUA quintile (Q5) was not associated with decline. No associations were observed for executive function, verbal fluency, or psychomotor speed. In males, no significant associations between SUA levels and change in cognitive function were observed.

**Conclusion:**

Low SUA levels were linked to decline in the measure of global cognition and episodic memory among females but not males. High SUA levels were not associated with cognitive decline. Managing SUA levels within the physiological range may support cognitive health, particularly in older females.

## Introduction

As life expectancy increases and more people are living past age 65, cognitive dysfunction is becoming an increasingly significant health challenge for older adults.^1^ With ageing, individuals may experience varying degrees of decline across multiple cognitive domains, including memory, attention, language, executive function, and visuospatial function.^2^^,^^3^

Serum uric acid (SUA), a by-product of purine metabolism, has a dual role in cognition.^4^^,^^5^ Its pro-oxidant properties promote free radical production, and cause inflammation, both of which are considered key pathogenic mechanisms underlying cognitive dysfunction.^6^ Conversely, the antioxidant properties help mitigate oxidative stress, potentially slowing neurodegenerative processes and delaying the onset of cognitive dysfunction.^7^^,^^8^

Associations between SUA levels and cognitive function have been examined in several prospective cohort studies, but with mixed findings. Some recent studies have linked higher levels of SUA to better cognitive performance in global cognition, attention, episodic memory, executive function, and mental intactness.^9-12^ Few studies, however, have reported an association between higher levels of SUA and poorer function in global cognition, executive function, language and memory,^13^^,^^14^ while others found no association.^15^^,^^16^

SUA levels differ by sex due to hormonal influences and variations in renal excretion patterns.^17^^,^^18^ These biological differences support the need for sex-stratified analyses when examining the relationship between SUA and cognitive function. However, sex-specific associations have been less frequently explored, and existing findings remain inconsistent. A study by Kueider et al.^19^ examining various cognitive domains reported no significant associations in either sex, except among males, for whom higher SUA was linked to better performance in attention, and visuospatial abilities. A study by Baena et al.^20^ assessed different cognitive domains and reported that higher levels of SUA were associated with better performance in executive function, but only in males. In contrast, a study by Lee et al.^21^ reported that higher levels of SUA were associated with better performance in global cognition, memory, executive function, and language among females’ patients with mild cognitive impairment (MCI) but not males. A study by Vannorsdall et al.^5^ conducted exclusively in female participants reported that higher levels of SUA were related to poorer memory performance, while no significant associations were observed for others domains. Most previous studies in this area have focused on younger or middle-aged adults across broad age ranges, with relatively limited research involving older adults. Additionally, several studies included both cognitively normal and impaired individuals in their analyses. The inclusion of younger or middle-aged adults may introduce heterogeneity, as their cognitive trajectories differ significantly from those of older adults, who are more susceptible to age-related cognitive changes. This variability can attenuate associations specific to ageing and make it difficult to draw conclusions relevant to older adults.^2^^,^^22^

In the current study, we aimed to assess the longitudinal association between baseline SUA levels and change in cognitive function among older adults over a median follow-up of 9 years.

## Methods

### Study population

We used data from the ASPirin in Reducing Events in the Elderly (ASPREE) study, the details of which were published elsewhere.^23^^,^^24^ Briefly, ASPREE was a double-blind, randomised, placebo-controlled clinical trial of 19 114 community-dwelling older adults aged 70 years or older (65+ years for African American and Hispanic communities in the United States) from Australia and the United States. Participants were recruited between March 2010 and December 2014 and received either 100 mg aspirin or placebo daily, with a median follow-up period of 4.7 years (until June 2017) to investigate disability-free survival (survival free from persistent physical disability, dementia, or death).

Eligibility criteria at enrolment into the ASPREE trial included being free of known cardiovascular disease events, independence-limiting physical disability (according to basic activities of daily living), diagnosed dementia, and achieving a score of 78 or more on the Modified Mini-Mental State Exam­ination (3MS, global cognition assessment) during screening.^25^ The ASPREE eXTention (ASPREE-XT) is a post-trial extension annual follow-up study of ASPREE participants until 2024.^26^ A total of 11 878 Australian participants were provided blood samples in the Healthy Ageing Biobank^27^ at baseline (enrolment) and Year 3 for clinical chemistry biomarker measurements including SUA (eFigure S1). In this analysis, we only included participants who had baseline SUA data. Participants using antigout preparations at baseline (due to the lack of data on pretreatment SUA levels) were excluded, leaving 11 411 in the current analysis.

### Uric acid measurement

Non-fasting blood samples were collected at the local ASPREE Clinical Trial Centre or laboratory, a pathology provider or in an ASPREE mobile laboratory.^27^ Blood samples were processed within four hours of collection, and serum was stored at −80°C for future analyses. SUA was analyzed using a uricase/peroxidase colorimetric method (Abbott Alinity ci, Abbott Diagnostics, Australia) with a coefficient of variation of 0.38% at 0.23 mmol/L and 1.2% at 0.57 mmol/L. Samples were analyzed at the Alfred Hospital Pathology Service (Melbourne, Australia), which is accredited by the National Association of Testing Authorities (NATA), adhering to the standards outlined in ISO 15189.^28^ For this analysis, SUA levels were converted to mg/dl by multiplying values in mmol/L by 59.49 and categorized into quintiles. The middle quintiles (Q2-Q4) were used as reference instead of the lowest quintile (Q1). This approach allows for a more balanced comparison, because the middle quintiles encompass the expected typical physiological range for the general adult population^29-31^ and aligns with the hypothesis of a nonlinear relationship between SUA levels and cognitive function.^32^

### Cognitive assessment

Cognitive performance was assessed over a median of nine years (IQR: 6-11 years) of follow-up using four cognitive tests listed below. During the ASPREE clinical trial, assessments were conducted at baseline, year 1, 3, and 5 and/or at the final visit, which was precipitated by early cessation of the trial in June 2017. During the ASPREE-XT period, cognition was assessed with 3MS only, in the first year and then annually with all cognitive tests. For all tests, a higher score indicated better cognitive function.

1. Global cognition was assessed using the 3MS, with scores ranging from 0 to 100. The 3MS consists of 34 questions across 15 items that assess various cognitive domains, including verbal recall, verbal fluency, reasoning, attention, visual construction, and expressive language.^33^
2. Episodic memory was measured using the Hopkins Verbal Learning Test-Revised (HVLT-R) delayed recall, with scores ranging from 0 to12. In this test, participants were required to recall a list of 12 nouns after a 20-25 minute delay following the initial 3 presentations.^34^
3. The Controlled Oral Word Association Test (COWAT) measured executive function and verbal fluency by requiring participants to generate, in 1 minute, words beginning with the letter F. The minimum score was 0, with no upper limit.^35^
4. The Symbol Digit Modalities Test (SDMT) assessed psychomotor speed by requiring participants to match digits with abstract symbols using a reference key. Scores ranged from 0 to 110.^36^

Data collected at year 2 (<100) and year 13 (<300) were excluded from the analyses as very few participants had data available at these time points. This was due to cognitive assessments not being part of the measurement protocol in year 2 and too few participants reaching the year 13 follow-up at the time of data analysis.

While the majority of cognitive tests were conducted in-person, a small proportion (ranging from 0% to 26% across visits) of 3MS cognitive assessments were conducted by phone due to COVID ( eTable 1). For phone-based assessments, items requiring physical responses, including naming body parts, reading and obeying instructions, writing a spoken sentence, and understanding a three-stage command were omitted. This reduced the maximum possible 3MS score from 100 to 74. To ensure comparability between in-person and phone assessments, scores from phone assessments were rescaled for analysis by dividing the phone 3MS score by 74 and multiplying by 100.^37^^,^^38^

To investigate overall cognitive performance across the 4 cognitive tests, a composite cognitive score was generated.^39^ The first step involved creating *z*-scores to standardize each cognitive test score at each time point, calculated as:


$$“Z-\mathrm{score}\;\mathrm{at}\;\mathrm{time}\;t=\frac{\mathrm{Participants}\;\mathrm{score}\;\mathrm{at}\;\mathrm{time}\;t-\;\mathrm{sample}\;\mathrm{mean}}{\mathrm{Sample}\;\mathrm{standard}\;\mathrm{deviation}}”$$

The *z*-scores for each test were then summed to produce the composite score. Composite scores are commonly used in cognitive research for global cognition status, as they have less variability than individual cognitive test scores and can reduce floor and ceiling effects.^40^

### Baseline covariates

Potential baseline confounders were identified in advance based on their plausible association with SUA and cognitive function. These included age, years of education (<12 years or ≥12 years), smoking status (never, former, or current), alcohol consumption status (never, former, or current), body mass index [BMI; normal weight (<25 kg/m^2^), overweight (25 to <30 kg/m^2^) and obese (≥ 30 kg/m^2^)], diabetes mellitus, hypertension, chronic kidney disease (CKD), use of diuretics, frailty status, and depressive symptoms. Diabetes mellitus (DM) was defined as self-report of diabetes or fasting blood glucose of greater than or equal to 126 mg/dL or on pharmaceutical treatment for diabetes. Hypertension was defined as use of antihypertensive medications or systolic blood pressure greater than or equal to 140 mmHg or diastolic blood pressure greater than or equal to 90 mmHg. CKD was defined as an estimated glomerular filtration rate of <60 ml per minute per 1.73 m^2^ or a urine albumin: creatinine ratio of 3 or more. Frailty status was categorized as non-frail, pre-frail or frail according to the adapted Fried frailty criteria.^41^ Diuretic (at baseline) use was recorded as a dichotomous variable (yes/no) and was defined using Anatomical therapeutic chemical classification codes. Depressive symptoms were assessed using the Center for Epidemiologic Studies Depression scale (CES-D 10), with a score of ≥ 8 out of 30 indicating the presence of depressive symptoms.^42^

### Statistical analysis

Descriptive statistics were used to compare participants’ baseline characteristics across the SUA quintiles. Continuous variables were presented as median (interquartile range, IQR) or mean (standard deviation, SD) and frequency (percentage) for categorical variables, as appropriate. Linear mixed-effects models (LMMs) with maximum likelihood regression were used to measure the association between baseline SUA levels and change in cognitive function over time. Separate LMMs were used for the 4 cognitive tests and the composite cognitive score in the sex-stratified analysis. Three models were generated to assess the robustness of the findings:^1^ an unadjusted model to examine the crude association,^2^ a model adjusted for age to account for its potential confounding effect, and^3^ a fully adjusted model controlling for additional baseline covariates listed above. Additionally, CKD status and use of diuretic were included in model 3 for separate analysis as they are known to directly influence SUA levels rather than acting as confounders.^43^ Participant-specific random intercepts and slopes were included, as exploratory analysis suggested considerable variability between participants in baseline cognitive scores and trajectories. Interaction effects between baseline SUA levels and cognitive change over time (baseline; 0, annual visit; 1, 3, and 4 to 12) were also included to assess whether the rate of change in cognitive function over time differed between SUA quintiles and sex. Marginal effects plots were generated to visually illustrate the predicted cognitive function trajectories across SUA quintiles, providing an intuitive interpretation of the modeled associations. Results are presented as beta (β) coefficients with standard errors (SE) for the intercept (baseline) and change over time (baseline SUA × annual visit), along with corresponding *p*-values. Effect modification by sex was evaluated for all 4 cognitive functions using a three-way SUA × sex × time interaction in the mixed-effects models. Separate sensitivity analyses were conducted by (1) excluding those with CKD and those using diuretic at baseline, (2) excluding participants whose 3MS score were collected via phone, and (3) categorizing SUA levels into quartiles to examine whether the associations were driven by extreme SUA groups. All *p*-values were 2-sided, and a *p* < .05 was considered statistically significant. Analyses were performed using Stata software, release 17 (StataCorp LLC, College Station, TX).

### Ethics

The ASPREE study received multiple institutional review board approvals in the US and Australia. In Australia, primary ethics approval was granted by Monash University Human Research Ethics Committee [CF07/3730-2006/745MC], and ethics approval for the ASPREE Healthy Ageing Biobank was obtained from the Alfred Hospital Ethics Committee (18/08). All participants provided written informed consent before enrolment.

## Results

### Study participants

A total of 11,411 participants were included in the analysis, comprising 55.3% females with a median follow-up of 9 years. The median age was 73.8 years (IQR: 71.8-77.2) for males and 73.9 years (IQR: 71.7-77.5) for females. At baseline, 467 (3.9%) participants were excluded due to the use of anti-gout medications (eFigure S1). Tables 1 and 2 present the baseline descriptive characteristics of the participants, stratified by SUA quintiles, separately for males and females. In males, the median SUA levels were 6.27 mg/dL (IQR: 5.59-7.29) slightly higher than in females who had levels of 5.25 mg/dL (IQR: 4.41-6.1) and distributions were quite similar across the sex-strata (eFigure S2). History of smoking and alcohol consumption was more common among males (56.4% and 90.6%, respectively) than females (34.5% and 78.7%). Diabetes was more prevalent in males than females (11.4% vs 7.8%), whereas diuretic use was higher in females than males (27.7% vs 20.2%). Other baseline covariates, including age, education, BMI, frailty, CKD, and hypertension, were relatively similar between sexes. At baseline, females had better cognitive performance than males across all 4 cognitive tests. Participants in the highest SUA quintile (Q5), both males and females, had a higher prevalence of CKD, frailty, hypertension, diuretic use, and obesity, compared to the reference group (Q2-Q4). Additionally, females in the highest SUA quintile (Q5) were older and had a higher prevalence of diabetes than the reference group.

**Table 1.** Baseline characteristics of the males’ participants (*n* = 5097) by SUA quintiles.

	Overall	Q1	Q2-Q4	Q5
***n* (%)**	5097	1040 (20.4)	3076 (60.4)	981 (19.3)
**SUA (mg/dL), median (IQR)**	6.27 (5.59-7.29)	4.75 (4.41-5.08)	6.44 (5.93-6.95)	8.31 (7.8-8.81)
**Age (years) median (IQR)**	73.8 (71.6-77.2)	73.9 (71.6-77.6)	73.7 (71.6-77.1)	73.8 (71.8-76.8)
**Education (years), *n* (%)**				
** <12**	2883 (56.6)	562(54)	1744 (56.7)	404 (41.2)
** ≥12**	2214 (43.4)	478 (46)	1332 (44.3)	577 (58.8)
**Smoking status, *n* (%)**				
** Never**	2271(44.6)	503 (48.4)	1375 (44.7)	393 (40.1)
** Former**	2647 (52.9)	492 (47.3)	1590 (51.7)	565 (57.6)
** Current**	179 (3.5)	45 (4.3)	111 (3.6)	23 (2.3)
**Alcohol consumption, *n* (%)**				
** Never**	480 (9.4)	106 (10.2)	301(9.8)	73 (7.4)
** Former**	282 (5.5)	66 (6.4)	173 (5.6)	43 (4.4)
** Current**	4335 (85.1)	868 (83.4)	2602 (84.6)	865 (88.2)
**BMI categories, kg/m^2^, *n* (%)**				
** Under/normal weight (<25)**	1128 (22.1)	343 (33)	661(21.5)	124 (12.7)
** Overweight (25 to < 30)**	2722 (53.4)	541 (52)	1692 (55.0)	489 (49.9)
** Obese (≥30)**	1247 (24.5)	156 (15)	723 (23.5)	368 (37.5)
**Frailty status (fried), *n* (%)^a^**				
** Non-frail**	3306 (64.9)	682 (65.9)	2010 (65.3)	614 (62.6)
** Pre-frail**	1722 (33.8)	345 (33.2)	1033 (33.6)	344 (35.1)
** Frail**	69 (1.3)	13 (1.2)	33 (1.1)	23 (2.3)
**Diabetes, *n* (%)^b^**	579 (11.4)	124 (11.9)	338 (11.0)	117 (11.9)
**Hypertension, *n* (%)^c^**	3819 (74.9)	719 (69.1)	2274 (73.9)	826 (84.2)
**CKD, *n* (%)^d^**	1132 (24.0)	152 (15.7)	617 (21.7)	363 (39.8)
**Diuretics used, *n* (%)**	1028 (20.2)	129 (12.4)	559 (18.2)	340 (34.7)
**CES-D 10 (mean, SD)**	2.8 (3.1)	2.9 (3.1)	2.8 (3.1)	2.8 (3.0)
**Cognitive test score**				
** 3MS, mean (SD)**	92.9 (4.6)	93.1 (4.5)	92.9 (4.6)	92.7 (4.5)
**HVLT-R-delayed recall, mean (SD)**	7.2 (2.9)	7.3 (2.9)	7.3 (2.9)	7.2 (2.8)
**COWAT-letter *F*, mean(SD)**	11.5 (4.5)	11.7 (4.6)	11.5 (4.6)	11.3 (4.3)
**SDMT, mean (SD)**	35.9 (9.7)	36.1(9.6)	35.9 (9.7)	35.9 (9.7)

Abbreviations: 3MS, Modified Mini-Mental State Examination; BMI, body mass index; COWAT-F, single letter Controlled Oral Word Association Test; HVLT-R-Delayed recall, Hopkins Verbal Learning Test-Revised Delayed Recall; IQR, interquartile range; n, sample size; SD, standard deviation; SDMT, Symbol Digit Modalities Test; SUA, serum uric acid.

a Fried frailty phenotype, which reflects compromised functioning based on weight loss, exhaustion, slow gait speed, low grip strength, and low physical activity. Not frail is no compromised function, pre-frail is compromised functioning in one or 2 domains, and frail is compromised functioning in 3 or more domains.

b Diabetes (mellitus) was defined as self-report of diabetes or fasting blood glucose of greater than or equal to 126 mg/dL or on pharmaceutical treatment for diabetes.

c Hypertension was defined as use of antihypertensive medications or systolic blood pressure greater than or equal to 140 mmHg or diastolic blood pressure greater than or equal to 90 mmHg.

d CKD (chronic kidney disease) was defined as an estimated glomerular filtration rate of <60 mL per minute per 1.73 m^2^ or a urine albumin: creatinine ratio of 3 or more.

**Table 2.** Baseline characteristics of the females’ participants (*n* = 6314) by SUA quintiles.

	Overall	Q1	Q2-Q4	Q5
***n* (%)**	6314	1370 (21.7)	3791(60.0)	1153 (18.3)
**SUA (mg/dL), median (IQR)**	5.25 (4.41-6.1)	3.9 (3.56-4.07)	5.25 (4.75-5.76)	7.29 (6.78-7.8)
**Age (years) median (IQR)**	73.9 (71.7-77.5)	73.6 (71.5-77.0)	73.8 (71.7-77.4)	74.6 (71.9-78.4)
**Education (years), *n* (%)**				
** <12**	3953 (62.6)	802 (58.5)	2372 (62.6.6)	779 (67.6)
** ≥12**	2361 (37.4)	568 (41.5)	1419 (37.4)	374 (32.4)
**Smoking status, *n* (%)**				
** Never**	4132 (65.5)	891(65.1)	2491(65.7)	750 (65.1)
** Former**	2004 (31.7)	421(30.7)	1200 (31.7)	383(33.2)
** Current**	178 (2.8)	58 (4.2)	100 (2.6)	20 (1.7)
**Alcohol consumption, *n* (%)**				
** Never**	1348 (21.3)	298 (21.8)	760 (20.1)	290 (25.2)
** Former**	240 (3.8)	62 (4.5)	141(3.7)	37 (3.2)
** Current**	4726 (74.9)	1010 (73.7)	2890 (76.2)	826 (71.6)
**BMI categories, kg/m^2^, *n* (%)**				
** Under/normal weight (<25)**	1855 (29.4)	639 (46.6)	1065 (28.1)	151 (13.1)
** Overweight (25 to < 30)**	2501 (39.6)	536 (39.1)	1582 (41.7)	383 (33.2)
** Obese (≥30)**	1958 (31.0)	195 (14.3)	1144 (30.2)	619 (53.7)
**Frailty status (Fried), *n* (%)^a^**				
** Non-frail**	3996 (63.3)	896 (65.4)	2453 (64.7)	647 (56.1)
** Pre-frail**	2196 (34.8)	456 (33.3)	1270 (33.5)	470 (40.8)
** Frail**	122 (1.9)	18 (1.3)	68 (1.8)	36 (3.1)
**Diabetes, *n* (%)^b^**	492 (7.8)	71 (5.2)	270 (7.1)	151 (13.1)
**Hypertension, *n* (%)^c^**	4627 (73.3)	861 (62.9)	2776 (73.2)	990 (85.9)
**CKD, *n* (%)^d^**	1497 (25.6)	199 (15.6)	794 (22.6)	504 (47.2)
**Diuretics used, *n* (%)**	1750 (27.7)	232 (16.9)	952 (25.1)	566 (49.1)
**CES-D 10 (mean, SD)**	3.4 (3.4)	3.4 (3.5)	3.4 (3.4)	3.4 (3.2)
**Cognitive test score**				
**3MS, mean (SD)**	94.2 (4.2)	94.5 (4.2)	94.2 (4.2)	94.1 (4.2)
**HVLT-R-delayed recall, mean (SD)**	8.3 (2.7)	8.4 (2.8)	8.3 (2.7)	8.3 (2.6)
**COWAT-letter F, mean(SD)**	12.7 (4.6)	13.1 (4.7)	12.7 (4.6)	12.3 (4.3)
**SDMT, mean (SD)**	38.4 (9.8)	38.8 (10.1)	38.5 (9.7)	37.7 (9.7)

Abbreviations and notes are as defined in Table 1.

**Table 3.** Longitudinal cognitive change by baseline serum uric acid levels (mg/dl), stratified by sex: mixed effect linear regression model.

	Males (*n* = 5097)				Females (*n* = 6314)			
Cognitive function	Intercept		SUA × Time		Intercept		SUA × Time	
Global cognition (3MS)	β ± SE	*p*-value	β ± SE	*p*-value	β ± SE	*p*-value	β ± SE	*p*-value
SUA quintiles								
** Lowest (Q1)**	0.17 ± 0.14	.24	−0.04 ± 0.03	0.19	0.14 ± 0.12	0.24	−0.07 ± 0.03	.02
** Middle (Q2-Q4)**	Reference				Reference			
** Highest (Q5)**	−0.01 ± 0.14	.95	0.06 ± 0.04	0.07	0.15 ± 0.13	0.25	−0.03 ± 0.03	.30
**Episodic memory (HVLT-R: delayed recall)**								
** Lowest (Q1)**	0.07 ± 0.10	.48	−0.00 ± 0.01	0.89	0.03 ± 0.08	0.73	−0.03 ± 0.01	.02
** Middle (Q2-Q4)**	Reference				Reference			
** Highest (Q5)**	−0.06 ± 0.10	.56	0.01 ± 0.01	0.50	0.11 ± 0.09	0.20	0.00 ± 0.01	.99
**Executive function and verbal fluency (COWAT)**								
** Lowest (Q1)**	0.10 ± 0.15	.50	0.00 ± 0.02	0.84	0.19 ± 0.13	0.15	−0.02 ± 0.01	.30
** Middle (Q2-Q4)**	Reference				Reference			
** Highest (Q5)**	0.01 ± 0.15	.94	0.01 ± 0.02	0.68	−0.09 ± 0.14	0.55	−0.01 ± 0.02	.77
**Psychomotor speed (SDMT)**								
** Lowest (Q1)**	0.31 ± 0.29	.29	−0.04 ± 0.03	0.20	−0.00 ± 0.27	0.99	−0.05 ± 0.03	.08
** Middle (Q2-Q4)**	Reference				Reference			
** Highest (Q5)**	0.37 ± 0.30	.22	0.01 ± 0.03	0.75	0.19 ± 0.29	0.52	0.00 ± 0.03	.88
**Composite cognitive score**								
** Lowest (Q1)**	0.09 ± 0.08	.29	−0.00 ± 0.01	0.74	0.06 ± 0.07	0.36	−0.02 ± 0.01	.03
** Middle (Q2-Q4)**	Reference				Reference			
** Highest (Q5)**	0.01 ± 0.08	.86	0.01 ± 0.01	0.21	0.06 ± 0.07	0.40	−0.01 ± 0.01	.63

Abbreviations: 3MS, Modified Mini-Mental State Examination (3MS); COWAT, controlled oral word association test; HVLT-R, Hopkins verbal learning test revised-delayed recall; SDMT, Symbol Digit Modalities Test.

Composite cognitive score: overall cognition by summing *z*-scores of 3MS, HVLT-R delayed recall, COWAT and SDMT.

Model adjusted: age at randomization, baseline years of education, smoking status, alcohol consumption, 10-item Center for Epidemiologic Studies Depression Scale (CES-D 10), hypertension, diabetes, body mass index (BMI) and Fried frailty.

### Change in global cognition (3MS scores)

**Figure 1.**
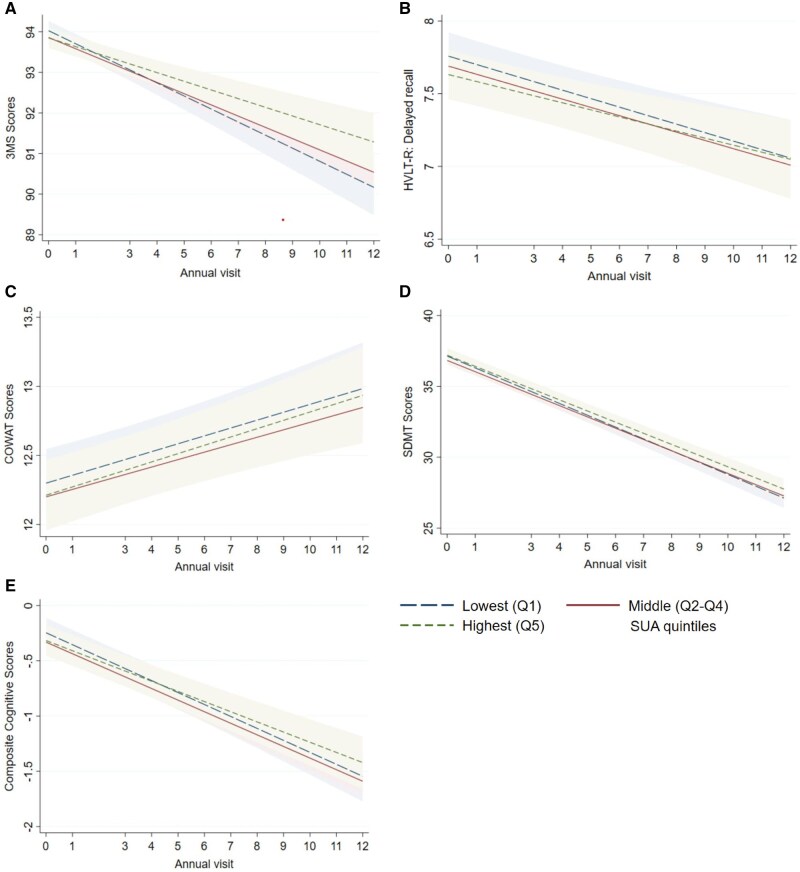
Estimated marginal means plot for males. Comparing lowest (Q1) and highest (Q5) quintiles of SUA levels with middle quintiles (Q2-Q4) to demonstrate the cognitive decline over time. (A: 3MS scores, B: HVLT-R delayed recall, C: COWAT scores, D: SDMT scores and E: composite cognitive scores). 95% confidence intervals are shown as shaded regions.

In the longitudinal analysis, males in the highest (Q5: β ±SE; 0.06 ± 0.04, *p *= .07) and the lowest (Q1: β ±SE; −0.04 ± 0.03, *p *= .19) SUA quintiles, showed no significant decline in the measure of global cognition compared to reference group (Table 3). In the marginal mean plots, all SUA categories showed a gradual decline in 3MS scores over time (Figure 1A). There were small differences in marginal means were observed across SUA categories over time (eTable S2), but there was no evidence that the average trajectory differed across SUA categories (substantial overlap in the 95% Cis; Figure 1A).

**Figure 2.**
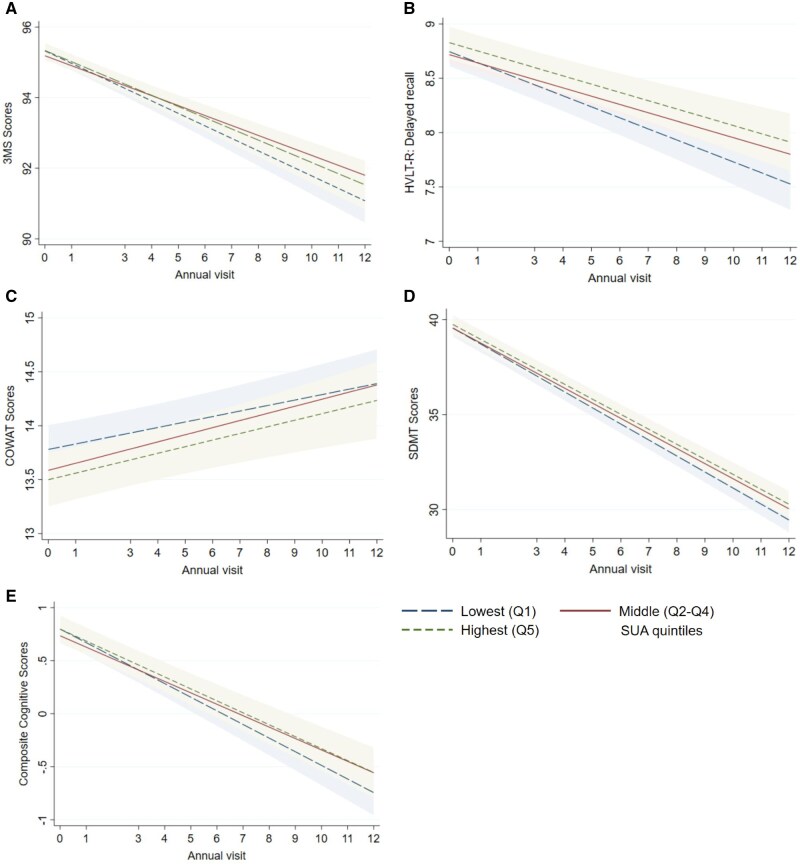
Estimated marginal means plot for females. Comparing lowest (Q1) and highest (Q5) quintiles of SUA levels with middle quintiles (Q2-Q4) to demonstrate the cognitive decline over time. (A: 3MS scores, B: HVLT-R delayed recall, C: COWAT scores, D: SDMT scores and E: composite cognitive scores). 95% confidence intervals are shown as shaded regions.

Among females, those in the lowest SUA quintile (Q1) had a mean (SD) baseline 3MS score of 94.5 (4.2) and demonstrated significant decline in performance (β ±SE = −0.07 ± 0.03, *p *= .02) over the follow-up period, compared to the reference group in the fully adjusted model. This trend is visually presented in Figure 2A, which shows a slightly higher decline in predicted mean 3MS scores over time for lowest quintile compared to middle quintiles. The association was not significant for those in the highest SUA quintile (Q5: β ±SE; −0.03 ± 0.03, *p *= .30) (Table 3 and Figure 2A). The three-way SUA × sex × time interaction was statistically significant for 3MS score (*p* = .001), indicating that the association between SUA and global cognition differed between males and females.

### Change in episodic memory (HVLT-R: delayed recall)

Over the follow-up period, males in the highest (Q5: β ±SE = 0.01 ± 0.01, *p *= .50) and the lowest (Q1: β ±SE = −0.00 ± 0.01, *p *= .89) SUA quintiles showed no significant decline in the measure of episodic memory compared to the reference group (Table 3). In the marginal mean plots, all SUA categories demonstrated a gradual decline in HVLT-R; delayed recall scores over time (Figure 1B). There were small differences in marginal means were observed across SUA categories over time (eTable S2), but there was no evidence that the average trajectory differed across SUA categories (substantial overlap in the 95% Cis; Figure 1B).

Among females, those in the lowest SUA quintile (Q1) had a mean (SD) baseline HVLT-R score of 8.4 (2.8), showed a slight decline in scores over time (β ±SE = −0.03 ± 0.01, *p *= .02), compared to the reference group. This trend is visually presented in Figure 2B, which shows a decline in predicted mean HVLT-R; delayed recall scores over time for lowest quintile compared to middle quintiles (eTable S2). The association was not significant for those in the highest SUA quintile (Q5: β ±SE = 0.00 ± 0.01, *p *= .99) (Table 3). There were small differences in marginal means were observed across SUA categories over time (eTable S2). It also showed in the marginal mean plots that the highest SUA quintile demonstrated a slightly lower decline in HVLT-R; delayed recall scores compared with the reference group, but there was no evidence that the average trajectory differed across SUA categories (substantial overlap in the 95% Cis; Figure 2B). The three-way SUA × sex × time interaction was statistically significant for HVLT-R: delayed recall score (*p* = .006), indicating that the association between SUA and episodic memory differed between males and females.

### Change in executive function and verbal fluency (COWAT)

A median of 9 years of follow-up, both sexes in the highest (Q5: β ±SE = 0.01 ± 0.02, *p *= .68 for males, and: β ±SE = −0.01 ± 0.02, *p *= .77 for females) and the lowest (Q1: β ±SE = 0.00 ± 0.02, *p *= .84 for males, and β ±SE = −0.02 ± 0.01, *p *= .30 for females) SUA quintiles showed no significant decline in the measure of executive function and verbal fluency compared to the reference group (Table 3). In the marginal mean plots, COWAT scores showed a gradual increase over time across all SUA categories in both sexes (Figures 1C and 2C). This upward trend likely reflects a practice effect, whereby participants improve their performance with repeated exposure to the same cognitive task over multiple assessment, rather than true improvement in executive function or verbal fluency. The three-way SUA × sex × time interaction was statistically significant for COWAT score (*p* < .001), indicating that the association between SUA and executive function and verbal fluency differed between males and females.

### Change in psychomotor speed (SDMT)

Over time, both sexes in the highest (Q5: β ±SE = 0.01 ± 0.03, *p *= .75 for males, and: β ±SE = 0.00 ± 0.03, *p *= 0.88 for females) and the lowest (Q1: β ±SE = −0.04 ± 0.03, *p *= .20 for males, and: β ±SE = −0.05 ± 0.03, *p *= .08 for females) SUA quintiles showed no significant decline in the measure of psychomotor speed compared to the reference group (Table 3). In the marginal mean plots, SDMT scores showed a gradual decline over time across all SUA categories in both sexes (Figures 1D and 2D). There were minimal differences in marginal means were observed across SUA categories over time (eTable S2), and the trajectories were closely aligned, with no evidence of differences observed (substantial overlap in the 95% Cis; Figures 1D and 2D). The three-way SUA × sex × time interaction was statistically significant for SDMT score (*p* < .001), indicating that the association between SUA and psychomotor speed differed between males and females.

### Change in composite cognitive score

In this longitudinal analysis, males in the highest (Q5: β ±SE = 0.01 ± 0.01, *p *= .21) and the lowest (Q1: β ±SE = −0.00 ± 0.01, *p *= .74) SUA quintiles showed no significant decline in composite cognitive score over time, compared to the reference group (Table 3). However, in females, those in the lowest SUA quintile (Q1) showed slight decline over time (β ±SE = −0.02 ± 0.01, *p *= 0.03) compared to the reference group. The association was not significant for females in the highest SUA quintile (Q5: β ±SE = −0.01 ± 0.01, *p *= .63) (Table 3). In the marginal mean plots, composite cognitive scores demonstrated a gradual decline over time across all SUA categories for both sexes (Figures 1E and 2E). There were small differences in marginal means were observed across SUA categories over time (eTable S2), but there was no evidence that the average trajectory differed across SUA categories (substantial overlap in the 95% Cis; Figures 1E and 2E). The three-way SUA × sex × time interaction was statistically significant for composite cognitive score (*p* = .001), indicating that the association between SUA and composite cognitive score differed between males and females.

### Sensitivity analysis and stepwise model adjustments

In sensitivity analyses excluding participants with CKD and those using diuretic, males in the highest SUA quintile (Q5) showed significantly better performance in the measure of global cognition compared to the reference group (β ± SE = 0.12 ± 0.05, *p *= 0.01), while the results for females remained unchanged. Similarly, excluding these participants did not alter the findings for the HVLT-R, COWAT, and SDMT assessments in either sex (eTable S3).

Excluding participants whose 3MS data were collected through phone did not affect the global cognition results, which remained consistent with those observed in the fully adjusted models for both males and females (eTable S3).

Similar findings (as presented in Table 2) were observed across all three models for both males and females; the unadjusted model, the age-adjusted model, and the fully adjusted model, which additionally accounted for CKD and diuretic used as confounders (eTable S4), indicating that the results remained robust regardless of the model specification.

To further assess whether the observed associations were driven by extreme SUA groups, the fully adjusted model was rerun with SUA categorized into quartiles. Among females, neither the lowest (Q1) nor highest (Q4) quartile of SUA was associated with a decline in any cognitive function compared to the reference group (Q2-Q3) (eTable S5). However, among males, highest SUA quartile (Q4) were associated with better performance in the measure of global cognition (3MS: β ± SE = 0.08 ± 0.03, *p* = .004), episodic memory (HVLT-R: delayed recall, β ± SE = 0.02 ± 0.01, p = 0.03), and the composite cognitive score (β ± SE = 0.02 ± 0.01, *p* = .009) compared with the reference group (Q2-Q3) (eTable S5).

## Discussion

Our study found that females in the lowest quintile of SUA experienced a significant decline in the measure of global cognition and episodic memory, but not in executive function and verbal fluency or psychomotor speed, compared to the reference group. In contrast, the highest quintile of SUA was not associated with decline in any of these cognitive functions. Among males, no significant associations were observed between SUA levels and cognitive function. These findings suggest that low SUA levels may play a sex-specific role in cognitive function, potentially increasing the vulnerability to cognitive decline in older females.

Although the observed effect size in global cognition was modest in absolute terms, translating this estimate into a cognitively meaningful metric helps contextualize its clinical importance. The rate of 3MS decline in normal aging is not fixed and varies by age, education, cohort characteristics, and follow-up duration, with estimates ranging from approximately 0.2 to 0.8 points per year.^44-46^ Using 0.5 points per year as an approximate benchmark, the additional decline observed in the lowest SUA quintile (β = –0.07 points/year) corresponds to about 0.14 years of additional cognitive aging per year, or roughly 1.4 additional years of cognitive aging over 10 years. This effect is particularly notable given that it was observed in cognitively unimpaired individuals at baseline, and considering that the 3MS is a global cognitive screening tool with known ceiling effects in healthy populations. Even small declines may therefore signal early cognitive vulnerability, especially when sustained over time.

Previous prospective cohort studies examining global cognition, executive function, language, attention, episodic memory, and visuospatial abilities reported no significant associations between higher baseline SUA levels and cognitive decline in any of these domains for either sex.^15^^,^^19^^,^^21^ Although these studies varied in population characteristics, cognitive test batteries, and the reference values for SUA, the overall findings are comparable. However, none of the prior studies reported the sex-stratified associations between lower levels of SUA and cognitive decline. Consistent with our results, a prospective cohort study by Huang et al. reported that lower plasma uric acid levels were associated with cognitive decline in the general Chinese older adults.^11^ Although evidence in the current literatures for an association between higher SUA levels and cognitive decline remains mixed, our results highlight a potential sex-specific impact of lowest SUA levels, particularly in increasing the risk of decline among older females, a relationship that has received limited attention in previous research.

The potential link between low levels of SUA and poorer cognitive function may be explained by the complex physiological mechanisms of SUA in the biological system. It has a paradoxical relationship with neurodegenerative diseases due to its dual function in neurons.^47^ Under intracellular conditions, particularly during oxidative stress or in the presence of transition metals such as iron and copper, SUA can contribute to the generation of reactive oxygen species (ROS), especially superoxide ($O_{2}^{-}$).^6^^,^^48^  $O_{2}^{-}$ reacts with nitric oxide (NO) to form peroxynitrite (ONOO^−^); a highly reactive oxidant capable of damaging nearly all cellular structures.^6^ This process has been implicated in the pathology of several neurodegenerative diseases, including multiple sclerosis,^49^ Parkinson’s disease,^50^ and Alzheimer’s disease.^51^

In contrast, SUA also acts as a potent antioxidant, particularly in the extracellular environment. Under normal physiological conditions, it helps mitigate oxidative stress by scavenging $O_{2}^{-}$ and NO.^6^ The enzyme superoxide dismutase (SOD) further detoxifies $O_{2}^{-}$ by converting it into oxygen (O_2_) and hydrogen peroxide (H_2_O_2_), thereby preventing the formation of ONOO^−^. This protective mechanism may help slow neurodegenerative processes and delay the onset of cognitive dysfunction.^7^^,^^8^

Lower SUA levels have been linked to decreased total antioxidant capacity, limiting the ability to counteract oxidative stress.^47^ These mechanisms may help explain our findings, where females with lower SUA levels experienced a decline in global cognition and episodic memory, possibly due to reduced antioxidant capacity and increased vulnerability to oxidative damage. As previously noted, the typical physiological reference range of SUA in general population coincides with our reference group, and the majority of the ASPREE participants fell within this range. The lowest quintiles included the individuals near or below the lower end of the reference range. Therefore, these findings provide further evidence that maintaining SUA levels within the typical normal range may be beneficial for cognitive health, particularly in older females.

Sex-stratified associations between the lowest SUA quintiles and cognitive function in our study may be explained by underlying physiological and hormonal differences. One possibility is that females generally have lower SUA levels than males, partly due to the uricosuric effect of estrogen, which promotes uric acid excretion through renal clearance.^17^ As a result, older females may be more vulnerable to having SUA levels below the threshold required for effective antioxidant protection. This relative deficiency in antioxidant defense may increase vulnerability to oxidative stress and neuronal damage, contributing to cognitive decline. In contrast, males typically have higher SUA levels, which may offer a greater advantage against oxidative stress even in the lower quintiles, potentially mitigating the risk of cognitive impairment. Additionally, sex differences in brain structure, vascular risk profiles, and hormonal changes following menopause may further modulate the relationship between SUA and cognitive aging.^17^^,^^18^ For example, the cognitive impact of vascular risk factors, particularly elevated blood pressure and LDL cholesterol, appears to differ by sex. Females show greater vulnerability to cognitive decline in the presence of these risks.^52^ Inflammatory markers such as C-reactive protein and interleukin-6 may also confer sex-differential risk, potentially mediated by hormonal influences: estrogen generally exerts antiinflammatory and cerebrovascular-protective effects, while testosterone may enhance certain inflammatory responses under physiological conditions.^53^ Furthermore, lifestyle factors that influence SUA levels also differ by sex. For example, males generally consume more purine-rich foods and alcohol,^54^^,^^55^ both of which increase SUA levels. These differences may contribute to sex-specific SUA patterns and cognitive trajectories.

In sensitivity analyses using quartiles of SUA levels, males in the highest quartile (Q4) demonstrated better performance in the measure of global cognition (3MS) and episodic memory (HVLT-R: delayed recall); however, no significant associations were observed in females. The variation of the results between quintiles and quartiles of SUA levels, likely reflect the influence of how SUA was categorized, which can differently allocate participants across risk profiles for cognitive decline. Several previous studies have suggested a potential U-shaped or J-shaped association between SUA levels and cognitive outcomes.^11^^,^^56^^,^^57^ Considering this hypothesis, using SUA categorized into quintiles is more appropriate than alternative grouping methods (eg, tertiles or quartiles), as it allows a more detailed examination of possible nonlinear associations across the SUA distribution. This finer categorization enables clearer identification of trends or threshold effects that might be obscured when using fewer, broader categories.

Our results add important new insights to the existing literature on the association between SUA and cognitive function, particularly regarding how low SUA levels affect specific cognitive domains in females. Further, excluding participants on anti-gout preparations strengthens our analysis by reducing the confounding effects of medication-induced changes in SUA levels, ensuring the observed associations reflect the natural relationship between SUA and cognition in untreated individuals. Methodologically, the large sample size of participants age over 70 years and a median follow-up period of nine years, enhanced the ability to detect long-term changes across specific cognitive domains and across sexes individually. Additionally, the analyses adjusted for several known demographics and risk factors, and the overall data quality was high.^58^ Cognitive assessments were performed by trained staff following strict protocols for administration and scoring.

Several important limitations of our analyses should be considered. First, although all participants were cognitively intact at enrolment, preclinical neurodegeneration may have begun years earlier and potentially influenced SUA metabolism; therefore, reverse causality cannot be fully excluded. Furthermore, evidence indicates that SUA levels follow sex- and age-dependent temporal patterns, meaning that SUA is not biologically stable over time.^59^ Consequently, relying on baseline SUA measurements alone does not account for changes in SUA levels that may influence cognitive outcomes.

Second, a small proportion of participants completed the 3MS assessment by phone. However, sensitivity analyses excluding these participants showed consistent results, suggesting this did not affect the findings. Third, SUA levels were measured in a non-fasting stored blood sample, which may introduce variability due to dietary influences. Lastly, although females with the lowest quintile of SUA experienced statistically significant declines in global cognition and episodic memory compared to those in the middle quintiles, clinical significance of these impacts needs further assessed.

## Conclusion

The present findings indicate that low baseline SUA is linked to decline in the measure of global cognition and episodic memory among initially cognitively sound older females. This association was not observed in males. Additionally, high baseline SUA levels were not linked to decline in any cognitive functions in either sex. Managing SUA levels within the typical physiological range may support better cognitive health and potentially reduce the risk of cognitive decline, especially in females.

## Supplementary Material

glaf296_Supplementary_Data

## Data Availability

All individual participant data (re-identifiable) that underlie the results reported in this article are available upon request to qualified researchers without limit of time, subject to approval and a standard data sharing agreement. Details regarding requests to access the data are available through the study website (ASPREE.org). The data will then be made available through a web-based data portal safe haven at Monash University (Melbourne, Australia).
