# Supplementary material for: Serum uric acid levels and longitudinal change in cognitive function in older adults: a sex-stratified population-based study
Source: J Gerontol A Biol Sci Med Sci. 2025 Dec 30;81(3):glaf296. doi: 10.1093/gerona/glaf296 (PMC13458196; doi:10.1093/gerona/glaf296)
Supplement: Supplementary file 1 [file glaf296_supplementary_data.pdf]

Supplementary Materials

Title: Serum uric acid levels and longitudinal change in cognitive function in older adults: a sex-stratified population-based study

Contents **Page**

Contents	Page
eFigure 1. Flowchart diagram of the ASPREE study participants included in this study. SUA= Serum uric acid	2
eFigure 2. Histogram of SUA levels in males and females	3
eTable 1. Summary of participants completing 3MS assessments by visit and mode of administration.	4
eTable 2. Marginal mean for different cognitive performances across SUA categories by sex over the follow-up period.	5
eTable 3. Sensitivity analysis excluding participants who had CKD, used diuretic and had 3MS data collected via phone calls stratified by sex: [a] mixed effect linear regression model.	6
eTable 4. Longitudinal cognitive change by baseline SUA levels stratified by sex: [a] mixed effect linear regression model.	7-8
eTable 5: Longitudinal cognitive change by baseline SUA levels (mg/dl), stratified by sex: mixed effect linear regression model.	9

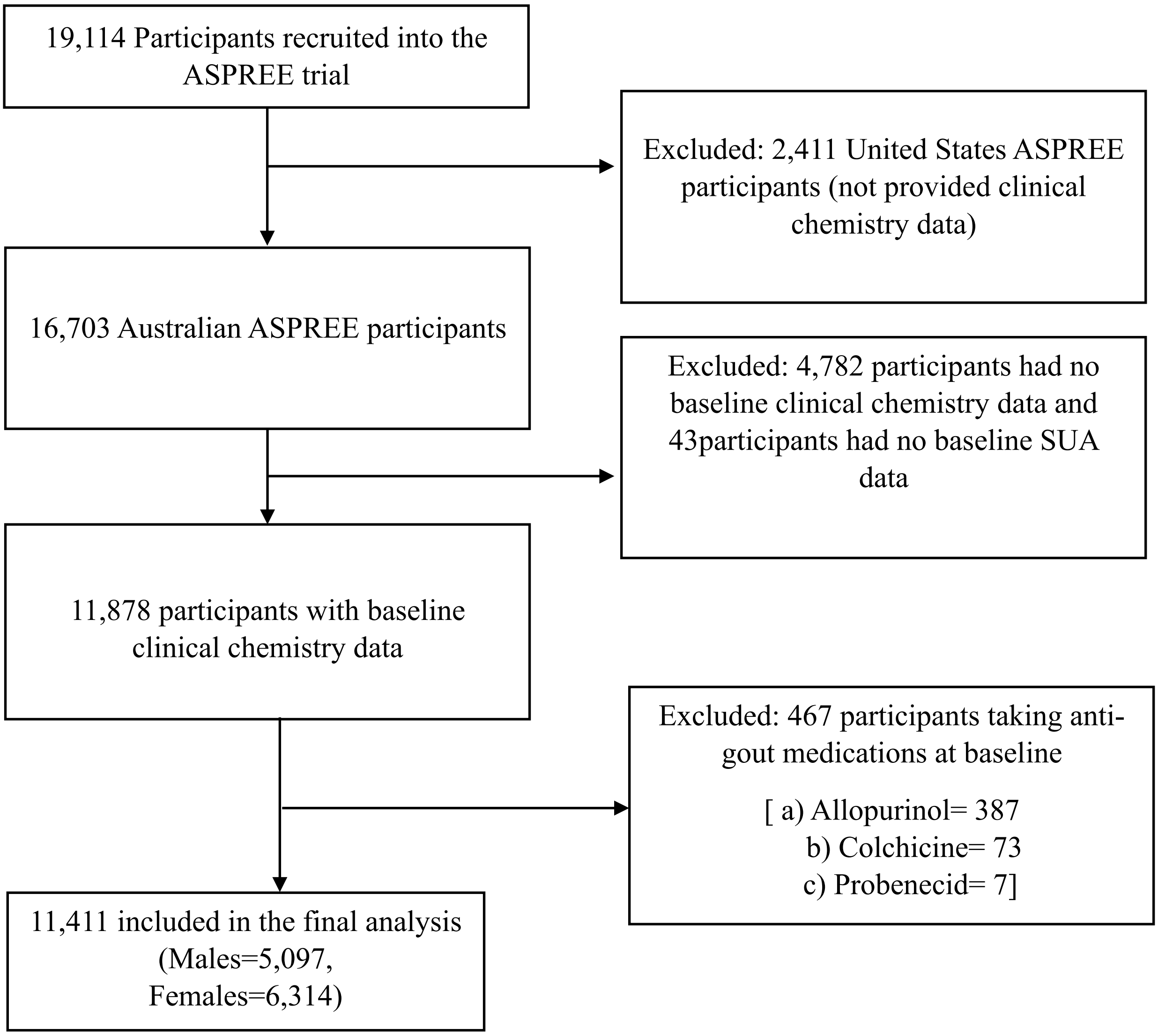

eFigure 1. Flowchart diagram of the ASPREE study participants included in this study. SUA= Serum uric acid

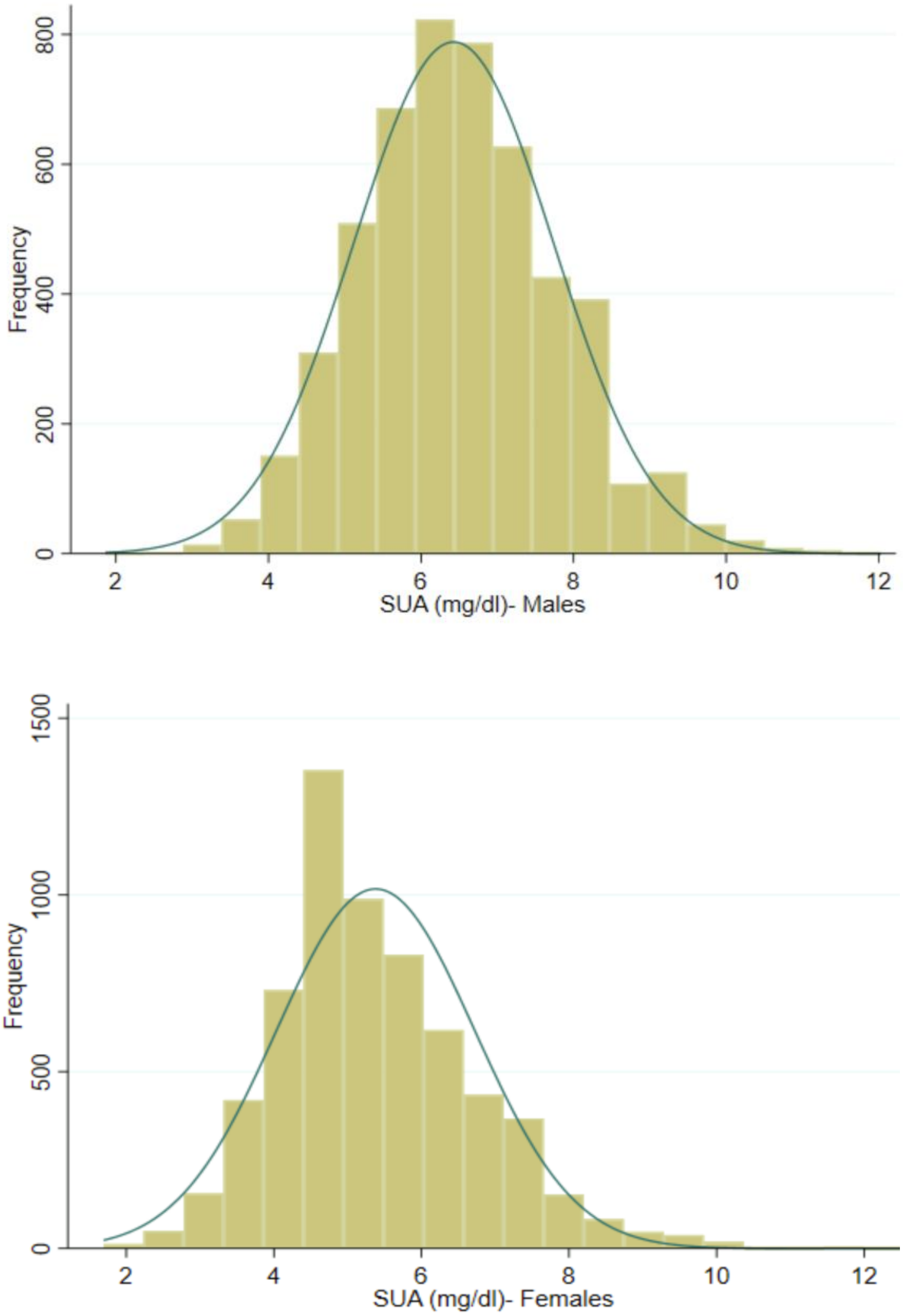

eFigure 2. Histogram of SUA levels in males and females

eTable 1. Summary of participants completing 3MS assessments by visit and mode of administration.

Visits	Total participants	In person	Phone call	Percentage (%)
Year-1	11,003	11,003	0	0
Year-3	10,122	10,122	0	0
Year-4	4,169	4,169	0	0
Year -5	8,871	8,771	100	1
Year -6	7,715	6,740	975	13
Year -7	7,631	5,935	1,696	22
Year -8	7,116	5,232	1,884	26
Year -9	6,708	4,942	1,766	26
Year -10	4,967	4,035	932	19
Year -11	3,203	2,971	232	7
Year -12	1,612	1,510	102	6

eTable 2. Marginal mean for different cognitive performances across SUA categories by sex over the follow-up period.

Males	3MS			HVLT-R			COWAT			SDMT			Composite scores		
Visits	Q1	(Q2-Q4)	(Q5)	Q1	(Q2-Q4)	(Q5)	Q1	(Q2-Q4)	(Q5)	Q1	(Q2-Q4)	(Q5)	Q1	(Q2-Q4)	(Q5)
Baseline	94.0	93.9	93.9	7.8	7.7	7.6	12.3	12.2	12.2	37.1	36.8	37.2	-0.3	-0.3	-0.3
Year-1	93.7	93.6	93.6	7.7	7.6	7.6	12.4	12.3	12.3	36.3	36.0	36.4	-0.4	-0.4	-0.4
Year-3	93.1	93.0	93.2	7.6	7.5	7.5	12.5	12.4	12.4	34.6	34.4	34.8	-0.6	-0.7	-0.6
Year-4	92.7	92.8	93.0	7.5	7.5	7.4	12.5	12.4	12.5	33.8	33.6	34.1	-0.7	-0.8	-0.7
Year -5	92.4	92.5	92.8	7.5	7.4	7.4	12.6	12.5	12.5	33.0	32.9	33.3	-0.8	-0.9	-0.8
Year -6	92.1	92.2	92.6	7.4	7.4	7.3	12.6	12.5	12.6	32.1	32.1	32.5	-0.9	-1.0.	-0.9
Year -7	91.8	91.9	92.4	7.4	7.3	7.3	12.6	12.6	12.6	31.3	31.3	31.7	-1.0	-1.0	-1.0
Year -8	91.5	91.6	92.1	7.3	7.2	7.2	12.8	12.6	12.7	30.5	30.5	30.9	-1.1	-1.2	-1.1
Year -9	91.1	91.4	91.9	7.2	7.2	7.2	12.8	12.7	12.8	29.6	29.7	30.1	-1.2	-1.3	-1.1
Year -10	90.8	91.1	91.7	7.2	7.1	7.2	12.9	12.7	12.8	28.8	28.9	29.3	-1.3	-1.4	-1.2
Year -11	90.5	90.8	91.5	7.1	7.1	7.1	12.9	12.8	12.9	28.0	28.1	28.5	-1.4	-1.5	-1.3
Year -12	90.2	90.5	91.3	7.1	7.0	7.1	13.0	12.9	12.9	27.1	27.3	27.8	-1.5	-1.6	-1.4
**Females**															
Baseline	95.3	95.2	95.3	8.7	8.7	8.8	13.8	13.6	13.5	39.6	39.6	39.8	0.8	0.7	0.8
Year-1	95.0	94.9	95.0	8.6	8.6	8.6	13.8	13.7	13.6	38.7	38.8	39.0	0.7	0.6	0.7
Year-3	94.3	94.3	94.4	8.4	8.5	8.6	13.9	13.8	13.7	37.0	37.2	37.4	0.4	0.4	0.5
Year-4	93.9	94.1	94.1	8.3	8.4	8.5	14.0	13.9	13.7	36.2	36.4	36.6	0.3	0.3	0.4
Year -5	93.6	93.8	93.6	8.2	8.3	8.5	14.0	13.9	13.8	35.4	35.6	35.8	0.2	0.2	0.2
Year -6	93.2	93.5	93.4	8.1	8.3	8.4	14.1	14.0	13.9	34.5	34.8	35.0	0.0	0.1	0.1
Year -7	92.9	93.2	93.1	8.0	8.2	8.3	14.1	14.1	13.9	33.7	34.0	34.2	-0.1	-0.0	0.0
Year -8	92.5	92.9	92.8	7.9	8.1	8.2	14.2	14.1	14.0	32.8	33.2	33.4	-0.2	-0.1	-0.1
Year -9	92.1	92.7	92.5	7.8	8.0	8.1	14.2	14.2	14.1	32.0	32.4	32.6	-0.4	-0.2	-0.2
Year -10	91.8	92.4	92.1	7.7	7.9	8.1	14.3	14.3	14.1	31.1	31.6	31.9	-0.5	-0.3	-0.3
Year -11	91.4	92.1	91.9	7.6	7.9	8.0	14.3	14.3	14.2	30.3	30.8	31.1	-0.6	-0.5	-0.4
Year -12	91.1	91.8	91.5	7.5	7.8	7.9	14.4	14.4	14.2	29.5	30.0	30.3	-0.7	-0.6	-0.6

Abbreviation: 3MS; Modified Mini-Mental State examination (3MS), HVLT-R; Hopkins Verbal Learning Test Revised -Delayed Recall, COWAT; Controlled Oral Word Association Test, SDMT; Symbol Digit Modalities Test

eTable 3. Sensitivity analysis excluding participants who had CKD, used diuretic and had 3MS data collected via phone calls stratified by sex: [a] mixed effect linear regression model.

Participants excluding CKD and use of diuretics (n=3234)								
	Males (n=3234)				Females (n=3620)			
Cognitive function	Intercept		$SUA_{baselien}$ x Time		Intercept		$SUA_{baselien}$ x Time	
Global cognition (3MS)<br>SUA (mg/dl), quintiles	β±SE	p	β±SE	p	β±SE	p	β±SE	p
Lowest (Q1)	0.17±0.16	0.29	-0.04±0.04	0.29	0.01±0.14	0.95	-0.08±0.03	0.01
Middle (Q2-Q4)	Reference							
Highest (Q5)	-0.11±0.21	0.59	0.12±0.05	0.01	-0.25±0.21	0.23	-0.03±0.05	0.60
Episodic Memory (HVLT-R: Delayed recall))								
Lowest (Q1)	0.08±0.11	0.47	0.001±0.01	0.92	-0.07±0.10	0.47	-0.02±0.10	0.05
Middle (Q2-Q4)	Reference							
Highest (Q5)	0.05±0.14	0.73	0.03±0.02	0.9	-0.06±0.14	0.69	0.01±0.02	0.69
Executive function and verbal fluency (COWAT))								
Lowest (Q1)	0.12±0.17	0.47	0.01±0.02	0.55	0.01±0.16	0.95	0.00±0.02	0.95
Middle (Q2-Q4)	Reference							
Highest (Q5)	0.05±0.22	0.83	-0.01±0.02	0.74	-0.64±0.24	0.01	0.02±0.03	0.43
Psychomotor speed (SDMT))								
Lowest (Q1)	0.13±0.34	0.71	-0.01±0.03	0.87	-0.27±0.33	0.41	-0.04±0.03	0.19
Middle (Q2-Q4)	Reference							
Highest (Q5)	0.15±0.43	0.73	0.04±0.04	0.40	-0.08±0.49	0.88	0.04±0.05	0.43
Composite cognitive score								
Lowest (Q1)	0.08±0.09	0.38	0.005±0.01	0.66	-0.05±0.08	0.52	-0.02±0.01	0.12
Middle (Q2-Q4)	Reference							
Highest (Q5)	0.03±0.12	0.81	0.03±0.01	0.06	-0.20±0.12	0.12	0.02±0.02	0.36
**Participants excluding 3MS data through phone call***								
Global cognition (3MS)	(n= 5097)				(n=6313)			
Lowest (Q1)	0.17±0.14	0.24	-0.04±0.03	0.19	0.11±0.12	0.33	-0.06±0.03	0.07
Middle (Q2-Q4)	Reference							
Highest (Q5)	-0.01±0.15	0.97	0.06±0.03	0.10	0.17±0.13	0.20	-0.03±0.03	0.36

(SUA quintiles: lowest=Q1, middle= (Q2-Q4) and highest = Q5)
Abbreviation: 3MS; Modified Mini-Mental State examination (3MS), HVLT-R; Hopkins Verbal Learning Test Revised -Delayed Recall, COWAT; Controlled Oral Word Association Test, SDMT; Symbol Digit Modalities Test
Composite cognitive score: overall cognition by summing *z*-scores of 3MS, HVLT-R delayed recall, COWAT, and SDMT.
[a] adjusted for age at randomisation, sex, and years of education, baseline smoking status, alcohol consumption status, 10-item Center for Epidemiologic Studies Depression Scale (CESD-10), hypertension, diabetes, BMI and Fried frailty. *Only 3MS had the phone call

eTable 4. Longitudinal cognitive change by baseline SUA levels stratified by sex: [a] mixed effect linear regression model.

**Males (n=5, 097)**												
**Cognitive function**	**Model 1**				**Model 2**				**Model 3**			
	**Intercept**		**SUA x Time**		**Intercept**		**SUA x Time**		**Intercept**		**SUA x Time**	
Global cognition (3MS)<br>SUA (mg/dl), quintiles	β±SE	p	β±SE	p	β±SE	p	β±SE	p	β±SE	p	β±SE	P
Lowest (Q1)	0.18±0.15	0.22	-0.05±0.03	0.19	0.24±0.15	0.10	-0.05±0.03	0.19	0.15±0.14	0.30	-0.04±0.03	0.19
Middle (Q2-Q4)	Reference				Reference				Reference			
Highest (Q5)	-0.11±0.15	0.47	0.06±0.04	0.08	-0.11±0.15	0.47	0.06±0.04	0.07	0.05±0.15	0.74	0.06±0.04	0.07
Episodic Memory (HVLT-R: Delayed recall)												
Lowest (Q1)	0.05±0.10	0.61	-0.002±0.01	0.87	0.10±0.10	0.30	-0.002±0.01	0.88	0.06±0.10	0.60	-0.001±0.01	0.89
Middle (Q2-Q4)	Reference				Reference				Reference			
Highest (Q5)	-0.11±0.10	0.28	0.009±0.01	0.48	-0.11±0.10	0.27	0.008±0.01	0.50	-0.03±0.10	0.78	-0.008±0.01	0.50
Executive function and verbal fluency (COWAT)												
Lowest (Q1)	0.16±0.15	0.29	0.003±0.02	0.83	0.18±0.15	0.22	0.003±0.02	0.83	0.09±0.15	0.54	0.003±0.02	0.84
Middle (Q2-Q4)	Reference				Reference				Reference			
Highest (Q5)	-0.008±0.15	0.60	0.007±0.02	0.66	-0.008±0.15	0.60	0.006±0.02	0.68	0.04±0.15	0.77	0.006±0.02	0.68
Psychomotor speed (SDMT)												
Lowest (Q1)	0.30±0.33	0.36	-0.04±0.03	0.20	0.52±0.31	0.10	-0.04±0.03	0.20	0.27±0.29	0.36	-0.04±0.03	0.20
Middle (Q2-Q4)	Reference				Reference				Reference			
Highest (Q5)	0.07±0.33	0.84	0.01±0.03	0.73	0.08±0.32	0.80	0.01±0.03	0.76	0.48±0.31	0.12	0.01±0.03	0.75
Composite cognitive score												
Lowest (Q1)	0.10±0.09	0.28	-0.004±0.01	0.71	0.15±0.09	0.09	-0.003±0.01	0.73	0.07±0.08	0.36	-0.003±0.01	0.74
Middle (Q2-Q4)	Reference				Reference				Reference			
Highest (Q5)	-0.07±0.09	0.46	0.01± 0.01	0.21	-0.07±0.09	0.45	0.01±0.01	0.21	0.05±0.08	0.55	0.01±0.01	0.21

eTable 4: (continued)

**Females (n=6,314)**												
**Cognitive function**	**Model 1**				**Model 2**				**Model 3**			
	**Intercept**		**SUA x Time**		**Intercept**		**SUA x Time**		**Intercept**		**SUA x Time**	
Global cognition (3MS)<br>SUA (mg/dl), quintiles	β±SE	p	β±SE	p	β±SE	p	β±SE	p	β±SE	p	β±SE	p
Lowest (Q1)	0.28±0.12	0.02	-0.07±0.03	0.03	0.22±0.12	0.07	-0.07±0.03	0.02	0.14±0.12	0.22	-0.07±0.03	0.02
Middle (Q2-Q4)	Reference				Reference				Reference			
Highest (Q5)	-0.20±0.13	0.14	-0.04±0.03	0.31	-0.07±0.13	0.57	-0.03±0.03	0.30	0.13±0.13	0.32	-0.03±0.03	0.30
Episodic Memory (HVLT-R: Delayed recall)												
Lowest (Q1)	0.08±0.08	0.36	-0.03±0.01	0.02	0.03±0.08	0.71	-0.02±0.01	0.02	0.02±0.08	0.76	-0.03±0.01	0.02
Middle (Q2-Q4)	Reference				Reference				Reference			
Highest (Q5)	-0.06±0.09	0.47	0.00±0.01	0.98	0.03±0.09	0.73	0.00±0.01	0.99	0.11±0.09	0.19	0.00±0.01	0.99
Executive function and verbal fluency (COWAT)												
Lowest (Q1)	0.42±0.14	0.002	-0.02±0.01	0.30	0.40±0.14	0.003	-0.02±0.01	0.30	0.19±0.13	0.15	-0.02±0.01	0.30
Middle (Q2-Q4)	Reference				Reference				Reference			
Highest (Q5)	-0.47±0.15	0.001	-0.005±0.02	0.77	-0.43±0.15	0.003	-0.005±0.02	0.76	-0.07±0.15	0.62	-0.004±0.02	0.77
Psychomotor speed (SDMT)												
Lowest (Q1)	0.33±0.29	0.27	-0.05±0.03	0.08	0.09±0.27	0.74	-0.05±0.03	0.08	-0.05±0.27	0.84	-0.05±0.03	0.08
Middle (Q2-Q4)	Reference				Reference				Reference			
Highest (Q5)	-0.74±0.31	0.02	0.005±0.03	0.87	-0.26±0.29	0.38	0.004±0.03	0.88	0.40±0.30	0.18	0.004±0.03	0.88
Composite cognitive score												
Lowest (Q1)	0.18±0.08	0.02	-0.02±0.01	0.04	0.13±0.07	0.08	-0.02±0.01	0.03	0.06±0.07	0.41	-0.02±0.01	0.03
Middle (Q2-Q4)	Reference				Reference				Reference			
Highest (Q5)	-0.21±0.08	0.01	-0.005±0.01	0.64	-0.11±0.08	0.17	-0.005±0.01	0.63	0.09±0.08	0.26	-0.005±0.01	0.63

(SUA quintiles: lowest=Q1, Middle= (Q2-Q4) and highest = Q5)
Abbreviation: 3MS; Modified Mini-Mental State examination (3MS), HVLT-R; Hopkins Verbal Learning Test Revised -Delayed Recall, COWAT; Controlled Oral Word Association Test, SDMT; Symbol Digit Modalities Test
Composite cognitive score: overall cognition by summing *z*-scores of 3MS, HVLT-R delayed recall, COWAT, and SDMT.
Model 1: unadjusted,
Model 2: adjusted for age at randomisation,
Model 3: Model 2+ baseline years of education, smoking status, alcohol consumption, CKD, diuretics, 10-item Centre for Epidemiologic Studies Depression Scale (CESD-10), hypertension, diabetes, BMI and Fried frailty.

eTable 5: Longitudinal cognitive change by baseline SUA levels (mg/dl), stratified by sex: mixed effect linear regression model.

**Cognitive function**	**Males (n=5,097)**				**Females (n=6,314)**			
	**Intercept**		**SUA x Time**		**Intercept**		**SUA x Time**	
Global cognition (3MS)	β±SE	p-value	β±SE	p-value	β±SE	p-value	β±SE	p-value
SUA quartiles								
Lowest (Q1)	0.14±0.19	0.46	-0.02±0.05	0.72	-0.07±0.10	0.51	-0.02±0.03	0.43
Middle (Q2-Q3)	Reference				Reference			
Highest (Q4)	-0.02±0.12	0.88	0.08±0.03	0.004	0.09±0.15	0.53	-0.03±0.04	0.50
Episodic Memory (HVLT-R: Delayed recall)								
Lowest (Q1)	0.16±0.13	0.21	0.01±0.02	0.46	0.01±0.07	0.87	-0.01±0.01	0.33
Middle (Q2-Q3)	Reference				Reference			
Highest (Q4)	0.05±0.08	0.55	0.02±0.01	0.033	0.02±0.10	0.88	0.01±0.01	0.29
Executive function and verbal fluency (COWAT)								
Lowest (Q1)	-0.06±0.19	0.76	0.02±0.02	0.38	0.21±0.12	0.08	-0.01±0.01	0.39
Middle (Q2-Q3)	Reference				Reference			
Highest (Q4)	-0.11±0.12	0.40	0.01±0.01	0.50	-0.11±0.16	0.51	0.02±0.02	0.34
Psychomotor speed (SDMT)								
Lowest (Q1)	-0.09±0.39	0.84	-0.03±0.04	0.20	0.06±0.24	0.81	-0.04±0.02	0.10
Middle (Q2-Q3)	Reference				Reference			
Highest (Q4)	0.32±0.25	0.20	0.02±0.03	0.43	0.27±0.33	0.42	0.02±0.04	0.65
Composite cognitive score								
Lowest (Q1)	0.04±0.10	0.72	0.01±0.01	0.61	0.04±0.06	0.51	-0.01±0.01	0.10
Middle (Q2-Q3)	Reference				Reference			
Highest (Q4)	0.02±0.07	0.75	0.02±0.01	0.009	0.03±0.09	0.76	0.01±0.01	0.53

Abbreviation: 3MS, Modified Mini-Mental State examination (3MS); HVLT-R, Hopkins Verbal Learning Test Revised-Delayed Recall; COWAT, Controlled Oral Word Association Test; SDMT, Symbol Digit Modalities Test, SUA, Serum Uric Acid

Composite cognitive score: overall cognition by summing *z*-scores of 3MS, HVLT-R delayed recall, COWAT and SDMT.

Adjusted model: age at randomisation, baseline years of education, smoking status, alcohol consumption, 10-item Center for Epidemiologic Studies Depression Scale (CES-D 10), hypertension, diabetes, BMI and Fried frailty.
